# Effects of the Appropriate Addition of Antioxidants from *Pinus densiflora* and *Mentha canadensis* Extracts on Methane Emission and Rumen Fermentation

**DOI:** 10.3390/ani10101888

**Published:** 2020-10-15

**Authors:** Shin Ja Lee, Ye Jun Lee, Jun Sik Eom, Hyun Sang Kim, You Young Choi, Seong Uk Jo, Suk Nam Kang, Ha Young Park, Do Hyung Kim, Sung Sill Lee

**Affiliations:** 1 Institute of Agriculture and Life Science & University-Centered Labs, Gyeongsang National University, Gyeongsangnam-do, Jinju-si 52828, Korea; tlswk1000@hanmail.net; 2 Division of Applied Life Science (BK21Plus), Gyeongsang National University, Gyeongsangnam-do, Jinju-si 52828, Korea; yejun0215@gmail.com (Y.J.L.); skyandstar07@naver.com (J.S.E.); 2437401@naver.com (H.S.K.); dudolboy301@naver.com (Y.Y.C.); jsu9412@naver.com (S.U.J.); 3 Department of Animal Resource, Daegu University, Gyeongbuk 38453, Korea; whitenightt@hanmail.net; 4 Department of Pathology, Busan Paik Hospital, Inje University College of Medicine, Busan 47392, Korea; hy08.park@gmail.com; 5 Department of Animal Science, Gyeongbuk Provincial College, Yecheon 36830, Korea; dh.kim@korea.kr

**Keywords:** *Pinus densiflora*, *Mentha canadensis*, methane emission, feed supplementation, ruminants

## Abstract

**Simple Summary:**

In the livestock industry, increasing attention is being focused on ways to reduce artificial greenhouse gas emissions from ruminants or provide alternatives to homeostasis. *Pinus densiflora* and *Mentha canadensis* extracts, with antioxidant properties, inhibit methanogenesis in ruminants and improve both digestion and growth of animals. Long-term stability of these plant extracts provides further support for their use as a substitute for other rumen regulators. It is expected that new additives for ruminants will be developed from *P. densiflora* and *M. canadensis* extracts, which are composed of phenolic compounds for improving the growth and immunity of ruminants.

**Abstract:**

This study aimed to investigate the optimal addition of terpene-based *Pinus densiflora* and *Mentha canadensis* extracts, with antioxidant and methane reduction effects, as feed supplements to ruminants. Two cannulated steers (450 ± 30 kg), consuming Timothy Hay and a commercial concentrate (60:40, *w/w*) twice daily (at 09:00 and 17:30) at 2% of body weight, with free access to water and a mineral block, were used as rumen fluid donors. In vitro fermentation experiments, with Timothy Hay as the substrate, were conducted with *P. densiflora* and *M. canadensis* extracts as supplements to achieve concentrations of 30, 50, and 70 mg/L on a Timothy Hay basis. *Fibrobacter succinogenes* decreased in proportion upon *P. densiflora* and *M. canadensis* extract supplementation at 50 mg/L, while the dry matter degradability of the feed was not significantly different (*p* < 0.05). Methane emission was significantly lower in the 50 and 70 mg/L treatment groups, for both extracts, at 12 h (*p* < 0.05). Based on methane production and antioxidant activity, our study suggests that 30 mg/L addition is the most appropriate level of supplementation.

## 1. Introduction

Methane produced from enteric fermentation in the livestock industry contributes approximately 20% of total global methane emissions [1]. Ruminal methanogenesis is an essential metabolic process that functions to maintain steady state fermentation, as it plays a vital role in scavenging the molecular hydrogen generated during fermentation. Methane is a natural by-product of ruminant digestion that serves as a hydrogen sink. However, given that methanogenesis is influenced by rumen microbial activity, the production of methane is dependent to varying extents upon animal species, age, and management, and, primarily, by the quality and quantity of feedstuffs administered to animals [2].

In the livestock industry, the market for feed additives is rapidly changing, with increasing attention being paid to ways of reducing artificial greenhouse gas emissions from ruminants or providing alternatives to homeostasis. Recently, several polysaccharides and plant extracts have been reported to show a prebiotic effect [3]. In China, various natural plants and extracts are used as animal feed additives for antimicrobial purposes, strengthening immunity, and reducing stress [4]. 

The components of *Pinus densiflora* and *Mentha canadensis* differ depending on region, climate, and other geographical and environmental features. *P. densiflora* is a needle-leaf tree distributed across eastern Asia. In recent years, pine needles have been reported to have antioxidant, antibacterial, anti-inflammatory, antimutagenic, and anti-tumor activities [5,6,7,8]. Several chemical compositions of pine needles have been identified to date [9]. *P. densiflora* is composed of 21.55% α-pinene, 6.36% camphene, 9.32% β-pinene, 0.46% myrcene, 13.05% limonene, 8.39% terpinolene, 1.07% α-terpineol, and 5.01% bornyl acetate [10]. The essential oils of *P. densiflora* have mild anti-microbial properties and can inhibit the growth of both Gram-positive and Gram-negative bacteria, as well as fungi [11]. Pine oil contains 58–97% β-ocimeneyne; almost all other components, such as piperitone (11.74%), camphene (7.20%), and β-pinene (5.64%), have been identified as monoterpene components. The ester component, endobornyl acetate, was analyzed to be present at approximately 5.63% [12].

*M. canadensis* is distributed all over the world and can be found in many environments. *M.* species, one of the world’s oldest and most popular herbs, and it is widely used in cooking and as an alternative or complementary therapy, mainly for the treatment of gastrointestinal disorders, such as flatulence, indigestion, nausea, vomiting, anorexia, and ulcerative colitis. The essential oils and extracts of *M.* species are documented to possess antimicrobial, fungicidal, antiviral, insecticidal, and antioxidant properties [13]. The main components of *M.* species are carbon (40.8 ± 1.23%) and limonene (20.8 ± 1.12%), followed by 1,8-cineole (17.0 ± 0.60%), β-pinene (2.2 ± 0.25%), cis-dihydrocarbon (1.9 ± 0.49%), dihydrocarveol (1.7 ± 0.31%), and α-pinene (1.4 ± 0.17%) [14].

Natural extracts of *P. densiflora* and *M. canadensis* are known to contain terpenoid substances, and extracts containing phenol and flavonoids are expected to be effective with respect to rumen fermentation and methane reduction. We aimed to verify the effect of these extracts on rumen fermentation. Furthermore, we evaluated their antibacterial and methane-reducing effects depending on the level of addition. Extracts *from P. densiflora* and *M. canadensis* were further evaluated for optimizing the amount to be added as a supplement in the ruminants’ functional feed.

## 2. Materials and Methods

### 2.1. Ethics Statement

All experimental procedures involving animals were approved by the Animal Ethics Committee of Gyeongsang National University (Gyeongsangnam-do, Jinju, Korea; GNU-180130-A0007).

### 2.2. In Vitro Batch Fermentation

Two Hanwoo steers (450 ± 30 kg), each fitted with a ruminal cannula, were used. Steers were offered free access to water and were fed 600 g/kg timothy and, 400 g/kg cracked corn-based concentrate (crude protein, 120 g/kg; ether extracts, 15 g/kg; crude fiber, 150 g/kg; crude ash, 120 g/kg; Ca, 7.5 g/kg; P, 9.0 g/kg; total digestible nutrients, 690 g/kg dry matter (DM) basis). The diet was fed to supply at 3% of BW in two equal portions at 07:00 and 1700 h daily, and intake adaptation periods were for a minimum of 14 days.

In vitro fermentation was conducted following the procedure described by Kim et al. [15]. Approximately 2 kg of ruminal fluid were collected from two ruminal cannulated steer 2 h after the morning feeding and were processed with a Waring blender under a CO_2_ atmosphere and filtered through four layers of cheesecloth and glass wool prior to combining with McDougall buffer [16]. The McDougall buffer and ruminal inoculums were combined to a 2:1 ratio, and this mixture was then added to fermentation vessels containing 0 or 300 mg (based on DM) of timothy as a substrate. *Pinus densiflora* and *Mentha canadensis* extracts for treatments were purchased from the Plant Extract Bank of Korea Research Institute of Bioscience and Biotechnology (Daejeon, Korea) and used in experiments. Prepared extracts of the 100 mg/mL stock solution used for treatments were diluted to 30, 50, and 70 mg/L with DMSO (Dimethyl sulfoxide, Sigma-Aldrich Chemical Co., St. Louis, MO, USA) through stepwise dilution.

The experimental design was completely randomized and conducted in duplicate and replicated on three separate days (*n* = 3 for statistical analyses). Gas production was monitored after 3, 6, 9, 12, 24, 48, and 72 h of incubation at 39 °C.

### 2.3. Total Phenolic and Flavonoid Content of Pinus densiflora and Mentha canadensis Extracts

Total phenolic content was determined with the Folin-Ciocalteu reagent according to the procedure described by Singleton and Rossi [17]. Briefly, Gallic acid was used as a reference standard calibration curve, and the results were expressed as milligram gallic acid equivalent (mg GAE)/g dry weight (g DW).

Total flavonoid content was determined using the method of Meda et al. [18] with minor modifications. Quercetin was used a standard calibration curve to quantify the total flavonoid content. Results were expressed in milligram quercetin equivalents (mg QE)/g dry weight (g DW).

The detailed procedures of such radical analyses were the same as those of Kang et al. [19].

### 2.4. Total DPPH, ABTS and HO, NO Radical Assay

The DPPH (2,2-Diphenyl-1-picrylhydrazyl) radical activity analysis was performed according to Kang et al. [19] method by modifying the method of Brand–Williams [20]. The free radical activity of samples 0.05 to 1 mg/mL in DMSO was measured. L-ascorbic acid was used as a standard. The absorbance was measured by a spectrophotometer (Ultrospec 2100 pro, Amersham Pharmacia Biotech Co., Piscataway, NJ, USA) at 517 nm.

The ABTS {2,2′-Azino-bis(3-ethylbenzothiazoline-6-sulphonic acid)} radical activity analysis was conducted using the method indicated by Re et al. [21]. Briefly, 2.85 mL of this ABTS+ solution was added to 0.15 mL of different concentrations of the samples, and the decrease in absorbance at 734 nm was observed after mixing for up to 10 min.

Hydroxyl (HO) radical scavenging activity of different concentrations of MEPA (*P. densiflora* and *M. Canadensis* extracts) was determined by the method of Elizabeth et al. [22]. The mixture’s absorbance was measured at 532 nm against an appropriate blank solution. BHA and Catechin was used as a reference compound. The results were compared with BHA and Catechin.

The Nitric oxide (NO) radical inhibition was estimated by the Griess Ilosvay reaction according to Hyoung [23] with slight modifications. Briefly, the absorbance was measured at 546 nm. Butylated hydroxyanisole was used as a standard.

The percent inhibition was calculated using the following formula: % inhibition = (A0 − A1)/A0 × 100.

In the formula, A0 is the absorbance of the control, and A1 is the absorbance of the sample and standard compound.

### 2.5. Analysis of In Vitro End-Products

At the end of each incubation time, the fermentation vessels were placed in an ice bath to measure the headspace gas pressure in each vessel using a digital readout voltmeter (Laurel Electronics, Inc., Costa Mesa, CA, USA). Gas samples for CH_4_ and CO_2_ analysis were transferred into a vacuum test tube (Vacutainer, Becton Dickinson, Franklin Lakes, NJ, USA) and analyzed by gas chromatography (Agilent Technologies HP 5890; Santa Clara, CA) using a TCD detector with a Column Carboxen 1006PLOT capillary column 30 m × 0.53 mm (Supelco), following the procedure described in Kim et al. [15]. A standard mixture of CH_4_ and CO_2_ (RIGAS, Daejeon, Korea) was used to determine the CH_4_ concentrations of samples. Vacuum tubes containing gas samples under refrigeration were warmed prior to gas analysis by allowing the tubes to equilibrate in a gas chromotograph (Agilent Technologies HP 5890; Santa Clara, CA, USA) for at least 30 min.

The amount of microbial growth rate was determined as optical density (OD) value at 550 nm with a spectrophotometer (Model 680, Bio-Rad Laboratories, Hercules, CA, USA). For measuring glucose, 200 μL of supernatant was mixed with 600 μL of DNS solution and incubated for 5 min in a boiling water bath. Glucose concentration was the OD at 595 nm, determined with a microplate reader (Model 680, Bio-Rad Laboratories, Irvine, CA, USA) [24].

### 2.6. Relative Quantification of Specific Ruminal Microbes

Quantitative real-time polymerase chain reaction was carried out in the same way as the papers of Lee et al. [24] and Lee at al. [25], Polymerase chain reaction (PCR) primer sets were then used in this study to detect and amplify DNA from *Fibrobacter succinogenes* (forward primer: GTT CGG AAT TAC TGG GCG TAA A; reverse primer: CGC CTG CCC CTG AAC TAT C), *Ruminococcus flavefaciens* (forward primer: CGA ACG GAG ATA ATT TGA GTT TAC TTA GG, reverse primer: CGG TCT CTG TAT GTT ATG AGG TAT TAC C), and *Ruminococcus albus* (forward primer: CCC TAA AAG CAG TCT TAG TTC G; reverse primer: CCT CCT TGC GGT TAG AAC A), and the primers used were the same as those referenced by Denman and McSweeney [26], Koike and Kobayashi [27], and Skillman et al. [28], respectively. A total bacteria primer set (forward: CGG CAA CGA GCG CAA CCC; reverse: CCA TTG TAG CAC GTG TGT AGC C) was used as the internal standard [25]. *Ciliate-associated methanogens* primer set (forward primer: AGG AAT TGG CGG GGG AGC AC; reverse primer: TGT GTG CAA GGA GCA GGG AC) was the same as those referenced by Luton et al. [29].

### 2.7. Statistical Analysis

Obtained data were analyzed using the general linear model procedure of SAS (version 9.2, SAS Inst. Inc., Cary, NC, USA) for a completely random design including terms for dosing levels, time, and their interaction. Duncan’s multiple range test was used to interpret any significant differences among the means values of doing levels for estimating the most appropriate level of supplementation. Orthogonal contrast was used to assess linear and quadratic relationships between the dosing levels of herb and pine and the dependent variables. Orthogonal coefficients for unequally spaced dosing levels were acquired using the IML procedure (SAS Inst. Inc., Greenwood Village, CO, USA). Means were considered significantly different if *p* ≤ 0.05 [30].

## 3. Results

### 3.1. Total Phenol and Flavonoid Contents, DPPH and ABTS Assays, OH and NO Scavenging Activities

The total phenol content in *P. densiflora* extracts was 925 ± 113.24 µg/g and that in *M. canadensis* extracts was 469 ± 14.27 µg/g. The total flavonoid content was 680.90 ± 67.49 and 715.77 ± 61.01 µg/g, respectively. DPPH was 49.38 ± 2.54 and 48.25 ± 1.28 at the concentration of 200 μg/mL, respectively, whereas ABTS was 78.85 ± 0.98 and 91.36 ± 0.29 at the concentration of 200 μg/mL, respectively.

The hydroxyl radical (OH)-scavenging activity of *P. densiflora* extract at concentrations of 200, 100, 50, and 10 μg/mL was 336.36 ± 100.39, 2251.17 ± 137.16, 1707.47 ± 115.93, and 790.53 ± 50.97, respectively. The nitric oxide (NO)-scavenging activity of *M. canadensis* extract at concentrations of 200, 100, 50, and 10 μg/mL was 54.93 ± 2.92, 32.50 ± 2.52, 20.35 ± 3.71, and 12.04 ± 3.74, respectively (Table 1).

### 3.2. Effect of Terpene-Based Plant Extracts on Rumen Fermentation and Dry Matter Degradability

The pH of *P. densiflora* and *M. canadensis* extracts was maintained in the range of 6.56–6.82 and 6.24–6.62, respectively. Among the treatments, the 50 mg/L treatment presented the lowest values (*p* < 0.05) at 6.48 and 6.69, respectively (herb: treatments effect, *p* = 0.0003, quadratic effect, *p* = 0.0099; pine: treatments effect, *p* = 0.0055, quadratic effect, *p* = 0.0338). Incubation for 12 h and 24 h showed a significant difference only in the 50 mg/L addition treatment (*p* < 0.05). Furthermore, the results of microbial growth had no adverse effect throughout the fermentation time. At 12 h, microbial growth was significantly lower (*p* < 0.05) than the control at *M. canadensis* 30 mg/L addition treatments. At 24 h, microbial growth was significantly lower (*p* < 0.05) than the control at *M. canadensis* 70 mg/L addition treatments. Glucose concentration was significantly lower than other treatments at *M. canadensis* 50 mg/L addition treatments after 12 h of incubation (treatments effect, *p* = 0.0244; quadratic effect, *p* = 0.0086). After 24 h, the control and *M. canadensis* 50 mg/L addition treatments were lower than the other treatments (treatments effect, *p* = 0.0242; quadratic effect, *p* = 0.0446). Protein concentration decreased by 70 mg/L of *M. canadensis* extract following 12 h (linear effect, *p* = 0.0273). The ammonia-nitrogen concentration changed linearly (*p* = 0.0273) following 12 h of incubation; this was decreased by 50 mg/L *M. canadensis* treatment after 12 h of incubation (Table 2).

Table 3 shows the effects of different *P. densiflora* and *M. canadensis* extracts on dry matter (DM) degradability and their parameters after the different incubation periods. There was no significant difference across treatments, although there was a pattern of DM degradability with time.

### 3.3. Effect of Terpene-Based Plant Extracts on Gas Profiles

Except for the 50 mg/L additive, *M. canadensis* extracts had a significantly higher effect on cumulative gas production (treatments effect, *p* = 0.0011). Methane emission was significantly higher (*p* < 0.05) at 50 mg/L of *M. canadensis* extract after 12 h of incubation (treatments effect, *p* = 0.0029). *Pinus densiflora* extracts showed significantly lower methane emission than the control, at 30 mg/L and 50 mg/L doses, after 12 h of incubation (treatments effect, *p* = 0.0024; linear effect, *p* = 0.0048). Carbon dioxide emission was higher in the 50 mg/L treatments than in the rest at 12 h (Table 4; herb: treatments effect, *p* = 0.0012, quadratic effect, *p* = 0.0116; pine: treatments effect, *p* = 0.0047; quadratic effect, *p* = 0.0553).

### 3.4. Effect of Terpene-Based Plant Extracts on VFA Profiles

The total volatile fatty acid (VFA) concentration under the 50 mg/L *M. canadensis* treatment was significantly higher than that of the control after 12 h of incubation (treatments effect, *p* < 0.0001; linear effect, *p* = 0.0001; quadratic effect, *p* < 0.0001). Acetate concentration after treatment with 50 mg/L *M. canadensis* extract was also significantly higher at 12 h of incubation (treatments effect, *p* < 0.0001; linear effect, *p* = 0.0019; quadratic effect, *p* = 0.0004). 

The propionate concentration of *M. canadensis* extract (50 mg/L) was higher than in other treatments (treatments effect, *p* = 0.0003; quadratic effect, *p* = 0.0015). The methane concentration under *P. densiflora* extract treatments (30 and 50 mg/L) was significantly lower than that under the control after 12 h of incubation (Table 5; treatments effect, *p* = 0.0049; linear effect, *p* = 0.0009).

### 3.5. Relative Quantification of Specific Ruminal Microbes

**Figure 1:**
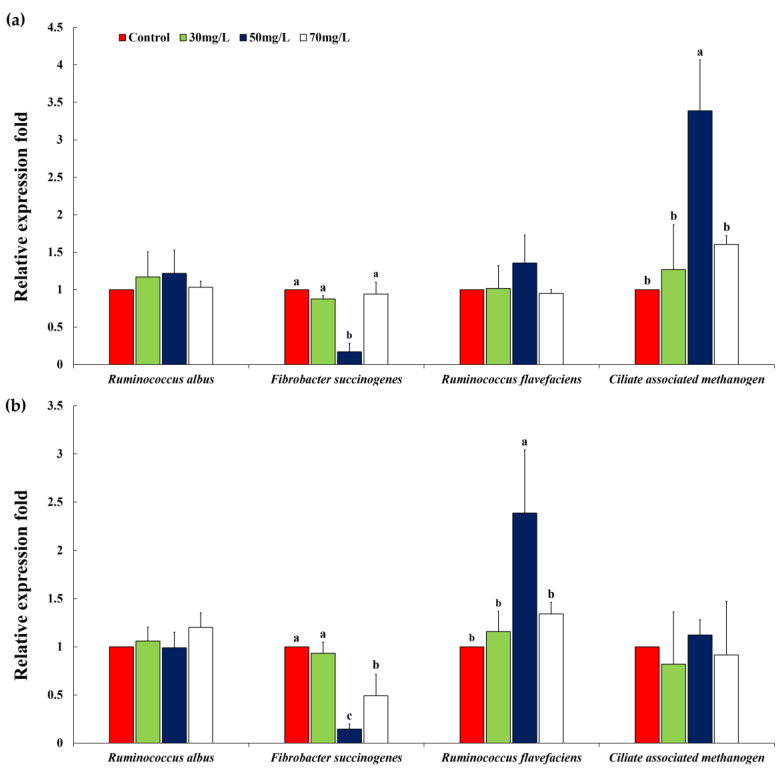
Relative quantification of in vitro rumen microbial population at (**a**) 12 h and (**b**) 24 h of incubation by *Pinus densiflora*. Error bars indicate the standard error of the mean (*n* = 3). ^a–c^ Means with different superscript letters in the same row indicate significant differences (*p* < 0.05).

*Pinus densiflora* extract, when added at 50 mg/L, remarkably reduced *Fibrobacter succinogenes* compared to the other treatments at 12 and 24 h (12 h: treatment effect, *p* = 0.0010, quadratic effect, *p* = 0.0025; 24 h: treatment effect, *p* = 0.0020, quadratic effect, *p* = 0.0085). However, *ciliate-associated methanogen* increased at 12 h (treatment effect, *p* = 0.0165; linear effect, *p* = 0.0155). The proportion of *Ruminococcus flavefaciens* was significantly higher (*p* < 0.05) under *P. densiflora* extract treatment (50 mg/L addition) than under other treatments at 24 h of incubation (Figure 1 and Table 6).

**Figure 2:**
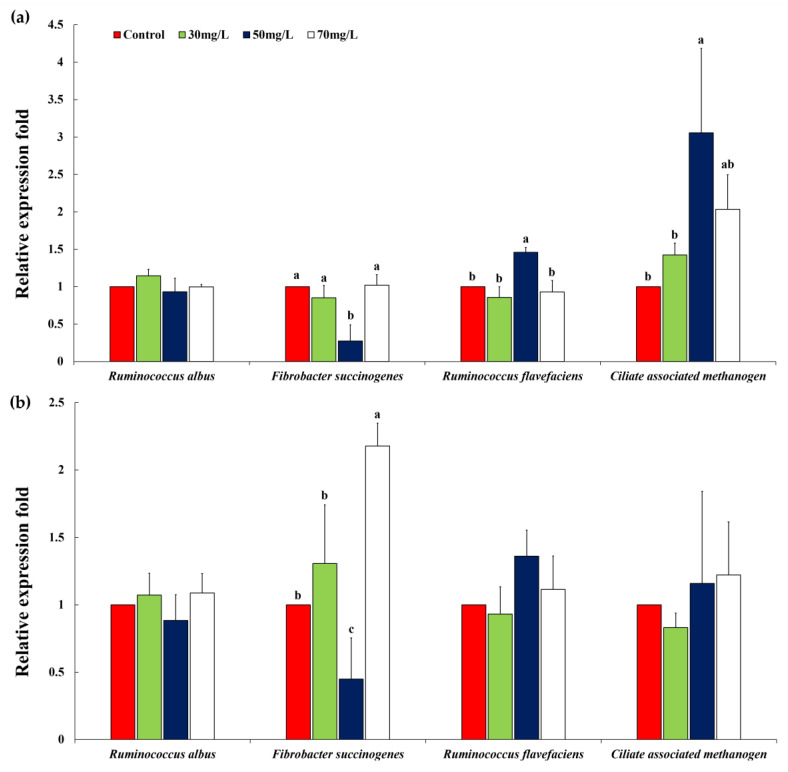
Relative quantification of in vitro rumen microbial population at (**a**) 12 h and (**b**) 24 h of incubation by *Mentha canadensis*. Error bars indicate the standard error of the mean (*n* = 3). ^a–c^ Means with different superscript letters in the same row indicate significant differences (*p* < 0.05).

*Mentha canadensis* extracts (50 mg/L treatments) also reduced *F. succinogenes* compared to other treatments at 12 h (treatment effect, *p* = 0.0001; linear effect, *p* = 0.0061; quadratic effect, *p* = 0.0004), although *ciliate-associated methanogen* increased at 12 h (treatment effect, *p* = 0.0008; linear effect, *p* = 0.0083; quadratic effect, *p* = 0.0192). The proportion of *F. succinogenes* was significantly lower (*p* < 0.05) than that under other treatments at 24 h (treatments effect, *p* = 0.0003; linear effect, *p* = 0.0003) (Figure 2 and Table 6).

## 4. Discussion

Polyphenolic compounds are secondary metabolites that are extensively distributed throughout the plant kingdom [31]. Polyphenol is a generic term used to encompass aromatic compounds that contain a hydroxyl group. Most polyphenol compounds, such as those in plant cell walls, polysaccharides, and lignin, including hydroxycinnamic acid, consist of ester bonds, or they exist as polymers [31,32]. In addition, they exhibit antioxidant properties by donating hydrogen via a hydroxyl group and stabilizing the resonance of the phenolic ring structure [33]. Total polyphenols and flavonoids influence antioxidant radical scavenging activity and are positively correlated [34,35], and such radical scavenging is generally determined using the DPPH radical and ABTS radical cation scavenging assays [18,19]. In macrophages, the inflammatory mediator NO plays an important role in killing bacteria or eliminating tumors; however, excessive NO formation owing to pathological causes could act as oxidative stress, causing cell damage, inflammation, and cancer [36]. In order to eliminate this oxidative stress, antioxidant and anti-inflammatory agents are important. Results of this study showed higher effects by *P. densiflora* extract than *M. canadensis* extract, which could be a very important factor in determining feed concentration. The pH range of rumen suitable for the digestion of high-fiber feed is 6.0–6.8 [37], and in this regard, the rumen pH range recorded in the present study was deemed to be adequate.

An analysis of the general components of *P. densiflora* revealed the following: moisture content, 53.48 ± 0.60%; crude protein content, 5.57 ± 0.86%; crude fat content, 5.16 ± 0.44%; crude ash content, 1.19 ± 0.20%; and crude fiber content, 11.32 ± 0.98% [38]. For *M. canadensis*, the water content was found to be 81.95 ±1.60%, crude protein content 1.25 ± 0.98%, crude fat content 0.40 ± 0.41%, ash content 2.05 ± 0.75%, and crude fiber content 2.48 ± 0.56% [39]. In this experiment, using a two extract from a raw material with a relatively high moisture content led to a composition that did not appear to be significantly affected by digestibility. There was, in fact, no significant difference in DM digestibility. This observation, despite the high content of the extract, will be an important finding in support of its use as an additive in economic terms. The total amount of gases, methane and carbon dioxide, was the highest when 50 mg/L extract was added. Increase in gas production is evidence of good fermentation. However, as the level of addition increased, the results were not proportional, perhaps because the added amount was beyond optimal or toxic.

Pine had not shown antibacterial activity in any of the strains studied, except for the weak antibacterial activity seen in the 2-mm clear zone at 10% concentration in *Pityrosporum ovale* [12]. In the present study, *P. densiflora* did not show any significant antibacterial activity. *F. succinogenes* levels suggested antibacterial activity when 50 mg/L extract was added, although the effect was not significant for the remaining microorganisms. Methane was reduced over the 12-h incubation period, owing to which the ciliate-associated methanogen may have decreased. However, in this experiment, it tended to increase in proportion to the amount of extract added. In particular, when 50 mg/L extract was added, it tended to increase explosively. We speculate that the observed increase in gas production could be ascribed to the limited negative effect of secondary metabolites, particularly flavonoids, in the extracts studied. These metabolites may have a positive influence on rumen fermentation, owing to their low to moderate amounts [40], or to the ability of rumen microorganisms to utilize these compounds as an energy source [41].

Microbial growth was significantly lower at 12 h in the case of 30 mg/L *M. canadensis* supplement. After 24 h of fermentation, it was significantly lower in the 70 mg/L supplementation group. Based on this, the optimal amount for the most effective microorganisms was identified as 50 mg/L. As in the PCR results described above, the number of microorganisms observed in 50 mg/L addition was high.

Oskoueian [42] previously reported that methane production, rumen protozoa, and methanogen populations reduced when 4.5% naringin and quercetin were added. Flavonoids significantly reduced methane concentration (*p* < 0.05), and total gas production was significantly reduced by flavones, myristin and kaempferol (*p* < 0.05). Rutin, naringin, and quercetin significantly increased total gas production (*p* < 0.05). Therefore, flavonoids have sufficient potential as additives [42]. In this experiment, there was no significant difference in methane production during the 24 h fermentation, and the effect of methane reduction was seen over a 12 h period. Considering that we used an extract from a plant rather than a purified flavonoid, the persistence was not especially high. In addition, unlike the experiment by Oskoueian [42] using 45 mg/L, methane emission showed a tendency to increase at 50 mg/L and decrease upon 30 mg/L and 70 mg/L additions. In in vitro batch culture for 12 h, methane emission under the 30 mg/L *M. canadensis* (*P. densiflora*) treatment was 13% (27%) compared with that under the control, showing methane reduction. However, as reported in earlier studies, the reduction effect was not persistent. Oskoueian [42] reported gas production to significantly decrease by the use of kaempferol, flavone, and myricetin (*p* < 0.05), whereas it significantly increased by the use of quercetin, naringin, and rutin (*p* < 0.05). Flavonoids were also reported to significantly inhibit methane production (*p* < 0.05). Aderao [43] recommended *Acacia nilotica* and *Ziziphus nummularia* leaves, which are rich in polyphenols, to be used as a feed as they reduce methane and improve green livestock production. Akanmu et al. reported that the use of crude extract (*Azadirachta indica*, *Moringa oleifera*, *Jatropha curcas*, *Tithonia diversifolia* and *Carica papaya*) as feed decreased methane production and increased digestibility [44].

As mentioned above, most previous studies have suggested that the addition of flavonoids or terpenoids are effective in rumen fermentation and methane reduction; however, the concentration of each addition was not confirmed. Therefore, the analysis of appropriate levels, as demonstrated in the present study, is valuable. Lowry and Kennedy reported an increase in the concentration of acetate and butyrate upon fermentation of quercetin, rutin, and naringin by rumen microorganisms [45]. However, in this experiment, propionate increased only at 12 h after 50 mg/L of *P. densiflora* and *M. canadensis* extracts were added.

Based on methane production or antibacterial activity, the current study revealed the most appropriate level of supplementation to be 30 mg/L. Many studies have been conducted on the ability to reduce methane, directly or indirectly, using flavonoids. Against Gram-positive bacteria, the production of propionate was increased compared to that of acetate in order to improve energy metabolism; such manipulation can contribute to increased efficiency of ruminant animals. This finding is similar to that of ionophores and represents antibacterial effects [45] Broudiscou et al. reported that *Equisetum arvense* extract and *Salvia officinalis* extract reduce methane by 1%–8% [46]. Another study using flavone, myricetin, and kaempferol demonstrated a reduced population of total bacteria, fungi, protozoa, and methanogens compared to that in control groups. Catechin reduced the total population of protozoan animals compared to that in the control group. Quercetin has been shown to reduce the total proportion of bacteria, fungi, protozoa, and methanogens in rumen [47].

Some flavonoids are effective in mitigating methane production and reducing acetate with propionates. However, the dose used in in-vitro experiments has been reported to be an additional dose that is not actually available in animal experiments and depends on the efficacy of a specific compound and response to its action [42]. Refined chemicals or synthetics would be more effective as feed for reducing methane; however, animals that have the feed would eventually be eaten by humans. Stability against chemicals has not yet been proven, and in-depth studies on relatively safer plant feed additives should be conducted in future. In conclusion, the effects of methane reduction and antioxidant properties could be confirmed by adding plant extracts containing phenol or flavonoids.

## 5. Conclusions

In this study, we confirmed the effects of plant extracts (derived from *P. densiflora* and *M. canadensis*) containing phenols and flavonoids on methane reduction and rumen fermentation. We found that the effects on methane emission were highest in those ruminants receiving *P. densiflora* and *M. canadensis* extracts at concentrations of 30 and 70 mg/L. With respect to rumen fermentation with different digestibility rates, we detected no difference between the effect of extracts supplemented at these two concentrations. Consequently, from an economic point of view, supplementing diets with a 30 mg/L extract would be considered the most beneficial. However, it is well established that additives and antibiotics of natural origin are more beneficial to animals than are their synthetic counterparts. Therefore, although the process is complex and time consuming, further research in this direction is warranted.

## Figures and Tables

**Table 1:** Antioxidant capacities (DPPH, ABTS, HO) and nitric oxide (NO) inhibition of *Pinus densiflora* and *Mentha canadensis* plant extracts.

Content	Terpene-Based Plant Extract	
	*Pinus densiflora*	*Mentha canadensis*
Total polyphenol content (µg GAE/g)	925 ± 113.24	469 ± 14.27
Total flavonoid content (µg QE/g)	680.90 ± 67.49	715.77 ± 61.01
Total antioxidant capacity		
DPPH		
200 (µg/mL)	49.38 ± 2.54	48.25 ± 1.28
100 (µg/mL)	43.27 ± 2.66	47.56 ± 1.63
50 (µg/mL)	21.90 ± 1.09	37.95 ± 4.63
10 (µg/mL)	16.63 ± 3.39	17.38 ± 1.53
ABTS		
200 (µg/mL)	78.85 ± 0.98	91.36 ± 0.29
100 (µg/mL)	67.34 ± 0.85	90.51 ± 0.68
50 (µg/mL)	52.20 ± 3.41	82.68 ± 1.20
10 (µg/mL)	23.11 ± 1.16	79.50 ± 1.97
HO		
200 (µg/mL)	2336.36 ± 100.39	299.97 ± 43.00
100 (µg/mL)	2251.17 ± 137.16	179.81 ± 30.84
50 (µg/mL)	1707.47 ± 115.93	102.46 ± 7.81
10 (µg/mL)	790.53 ± 50.97	37.11 ± 6.80
NO		
200 (µg/mL)	59.31 ± 1.36	54.93 ± 2.92
100 (µg/mL)	32.42 ± 2.01	32.50 ± 2.52
50 (µg/mL)	20.59 ± 3.14	20.35 ± 3.71
10 (µg/mL)	7.94 ± 2.59	12.04 ± 3.74

DPPH: 2,2-Diphenyl-1-picrylhydrazyl radical scavenging activity; ABTS: 2,2′-Azino-bis(3-ethylbenzothiazoline-6-sulphonic acid) radical scavenging activity; HO: Hydroxyl radical scavenging activity; NO: Nitric oxide (NO) inhibition; GAE: gallic acid equivalent; QE: quercentin equivalent.

**Table 2:** Effects of terpene-based plant extracts on ruminal fermentation characteristics after 12 and 24 h of in vitro incubation.

Incubation Time (h)	Treatment	Extract Concentration ^(1)^ (mg/L)				SEM ^(2)^	*p* Value ^(3)^		
		0	30	50	70		T	L	Q
pH									
12	Herb ^(4)^	6.58 ^a^	6.60 ^a^	6.48 ^b^	6.62 ^a^	0.01	0.0003	0.7580	0.0099
	Pine ^(5)^	6.79 ^a^	6.80 ^a^	6.69 ^b^	6.82 ^a^	0.02	0.0055	0.7644	0.0338
24	Herb	6.24	6.53	6.49	6.51	0.11	0.2922	0.1268	0.3111
	Pine	6.70 ^a^	6.73 ^a^	6.56 ^b^	6.71 ^a^	0.03	0.0136	0.2929	0.2027
Microbial growth rate, OD at 550 nm									
12	Herb	0.42 ^a^	0.30 ^b^	0.41 ^a^	0.39 ^ab^	0.03	0.0792	0.7914	0.0901
	Pine	0.34	0.34	0.40	0.35	0.06	0.8686	0.7922	0.7290
24	Herb	0.35 ^ab^	0.37 ^ab^	0.37 ^a^	0.31 ^b^	0.02	0.1179	0.2543	0.0433
	Pine	0.35	0.29	0.38	0.35	0.03	0.1862	0.5431	0.3383
Glucose (mg∙mL^−1^)									
12	Herb	0.07 ^a^	0.06 ^bc^	0.06 ^c^	0.07 ^ab^	0.00	0.0244	0.1329	0.0086
	Pine	0.06	0.08	0.06	0.05	0.01	0.2376	0.1892	0.1667
24	Herb	0.05 ^b^	0.07 ^a^	0.05 ^b^	0.06 ^ab^	0.01	0.0242	0.2306	0.0446
	Pine	0.06	0.06	0.05	0.05	0.01	0.3502	0.1135	0.4862
Protein (mg∙mL^−1^)									
12	Herb	0.20 ^a^	0.17 ^ab^	0.18 ^ab^	0.16 ^b^	0.01	0.0962	0.0273	0.8446
	Pine	0.20	0.20	0.23	0.21	0.01	0.1537	0.2807	0.4698
24	Herb	0.19 ^b^	0.19 ^b^	0.20 ^ab^	0.21 ^a^	0.01	0.0473	0.0105	0.2879
	Pine	0.22	0.22	0.23	0.21	0.01	0.3240	0.7081	0.1946
Ammonia nitrogen (mg∙mL^−1^)									
12	Herb	3.18 ^a^	3.93 ^a^	1.18 ^b^	2.93 ^a^	0.44	0.0123	0.1272	0.6570
	Pine	3.98	7.78	4.16	6.33	1.15	0.1324	0.4231	0.3784
24	Herb	2.60 ^ab^	2.27 ^b^	4.31 ^a^	2.89 ^ab^	0.52	0.0983	0.2542	0.5618
	Pine	4.80 ^b^	4.78 ^b^	5.51 ^ab^	6.53 ^a^	0.46	0.0854	0.0260	0.1910

^(1)^ Extract concentrations are based on quantity of Timothy Hay (300 mg) substrate. ^(2)^ SEM, Standard error of the mean. ^(3)^ T, Treatments effect; L, Linear effect; Q, Quadratic effect. ^(4)^ Herb: *Mentha canadensis* plant extract. ^(5)^ Pine: *Pinus densiflora* plant extract. ^a–c^ Means with different superscript letters in the same row indicate significant differences (*p* < 0.05).

**Table 3:** Effects of terpene-based plant extracts on dry matter (DM) degradability after 72 h of in vitro incubation.

Incubation Time (h)	Treatment	Extract Concentration ^(1)^ (mg/L)				SEM ^(2)^	*p* Value ^(3)^		
		0	30	50	70		T	L	Q
DM degradability, %									
3	Herb ^(4)^	19.33	18.67	17.89	19.56	0.76	0.4468	0.8850	0.1900
	Pine ^(5)^	22.11	21.22	23.00	21.33	1.29	0.7519	0.9133	0.8601
6	Herb	20.11	19.78	19.00	19.67	1.14	0.9159	0.6732	0.7375
	Pine	23.00	22.00	23.11	22.22	0.82	0.7196	0.7093	0.8722
9	Herb	21.44	21.22	21.22	20.00	1.01	0.7416	0.3842	0.5983
	Pine	23.00	23.89	23.44	23.11	1.14	0.9444	0.9673	0.5882
12	Herb	27.11	26.33	27.11	27.33	0.38	0.332	0.5261	0.1647
	Pine	29.89	29.78	29.11	31.00	0.85	0.505	0.5529	0.2887
24	Herb	30.78	33.78	34.67	29.44	2.00	0.2816	0.8716	0.0784
	Pine	32.44	33.22	31.44	33.33	1.25	0.6992	0.8741	0.7543
48	Herb	41.22	38.33	39.78	33.78	1.77	0.0776	0.0314	0.3655
	Pine	36.89	37.67	39.11	36.56	0.97	0.3167	0.8298	0.1564
72	Herb	49.37	52.46	50.79	50.96	1.43	0.5362	0.5485	0.3099
	Pine	48.67	48.56	55.22	51.89	1.63	0.0594	0.0568	0.6463

^(1)^ Extract concentrations are based on quantity of Timothy Hay (300 mg) substrate. ^(2)^ SEM, Standard error of the mean. ^(3)^ T, Treatments effect; L, Linear effect; Q, Quadratic effect. ^(4)^ Herb: *Mentha canadensis* plant extract. ^(5)^ Pine: *Pinus densiflora* plant extract.

**Table 4:** Effects of terpene-based plant extracts on gas profiles after 12 and 24 h of in vitro incubation.

Incubation Time (h)	Treatment	Extract Concentration ^(1)^ (mg/L)				SEM ^(2)^	*p* Value ^(*3*)^		
		0	30	50	70		T	L	Q
Total gas, mL/g DM									
12	Herb ^(4)^	190.27 ^b^	189.64 ^b^	204.58 ^a^	187.32 ^b^	2.01	0.0011	0.4233	0.0094
	Pine ^(5)^	167.94 ^ab^	163.24 ^bc^	175.01 ^a^	159.49 ^c^	2.37	0.0088	0.2539	0.0950
24	Herb	212.13	207.75	214.03	205.00	4.75	0.5535	0.4962	0.6923
	Pine	176.97 ^b^	177.92 ^b^	187.32 ^a^	176.97 ^b^	2.79	0.0811	0.4221	0.1367
Methane, mL/g DM									
12	Herb	10.83 ^b^	9.40 ^b^	13.33 ^a^	9.49 ^b^	0.54	0.0029	0.9572	0.1585
	Pine	11.95 ^a^	8.68 ^b^	11.13 ^a^	8.36 ^b^	0.51	0.0024	0.0048	0.5125
24	Herb	19.33	16.84	18.39	17.24	1.10	0.4242	0.3173	0.5270
	Pine	17.22	12.90	19.83	11.30	3.23	0.2955	0.4683	0.6308
Carbon dioxide, mL/g DM									
12	Herb	61.30 ^b^	64.26 ^b^	88.00 ^a^	64.66 ^b^	3.20	0.0012	0.0348	0.0116
	Pine	45.47 ^b^	41.36 ^bc^	52.44 ^a^	37.38 ^c^	2.06	0.0047	0.2098	0.0553
24	Herb	89.69	93.75	99.07	94.21	4.37	0.5405	0.3307	0.4248
	Pine	56.73	50.79	57.75	48.01	4.72	0.4465	0.3809	0.7546

^(1)^ Extract concentrations are based on the quantity of Timothy Hay (300 mg) substrate. ^(2)^ SEM, Standard error of the mean. ^(3)^ T, Treatments effect; L, Linear effect; Q, Quadratic effect. ^(4)^ Herb: *Mentha canadensis* plant extract. ^(5)^ Pine: *Pinus densiflora* plant extract. ^a–c^ Means with different superscript letters in the same row indicate significant differences (*p* < 0.05).

**Table 5:** Effects of terpene-based plant extract on VFA profiles after 12 and 24 h of in vitro incubation.

Incubation Time (h)	Treatment	Extract Concentration ^(1)^ (mg/L)				SEM ^(2)^	*p* Value ^(3)^		
		0	30	50	70		T	L	Q
Total VFA, mM									
12	Herb ^(4)^	53.85 ^c^	54.74 ^b^	59.77 ^a^	54.79 ^b^	0.27	<0.0001	0.0001	<0.0001
	Pine ^(5)^	53.98	60.36	59.37	55.70	1.95	0.1458	0.4766	0.0339
24	Herb	62.40	63.15	65.17	62.46	1.50	0.5527	0.6974	0.3463
	Pine	61.52	60.88	64.91	62.49	1.22	0.1815	0.2500	0.7364
Acetate, mM									
12	Herb	36.58 ^b^	36.90 ^b^	40.80 ^a^	37.04 ^b^	0.28	<0.0001	0.0019	0.0004
	Pine	36.20	39.68	39.22	36.40	1.79	0.4255	0.8482	0.1172
24	Herb	42.62	42.91	44.49	42.60	0.91	0.4475	0.6737	0.3417
	Pine	40.90	40.99	42.46	41.48	0.93	0.6371	0.4530	0.7138
Propionate, mM									
12	Herb	10.56 ^b^	10.77 ^b^	11.88 ^a^	10.61 ^b^	0.13	0.0003	0.0367	0.0015
	Pine	10.93 ^b^	11.74 ^a^	11.92 ^a^	10.92 ^b^	0.17	0.0049	0.5144	0.0009
24	Herb	12.59	12.64	13.28	12.47	0.36	0.4306	0.8262	0.3357
	Pine	12.52 ^b^	12.04 ^b^	13.44 ^a^	12.17 ^b^	0.28	0.0269	0.7993	0.3740
Butyrate, mM									
12	Herb	6.71	7.07	7.09	7.14	0.21	0.4762	0.1712	0.5345
	Pine	6.84	8.94	8.23	8.38	0.48	0.0750	0.0744	0.0800
24	Herb	7.20 ^b^	7.59 ^a^	7.41 ^ab^	7.40 ^ab^	0.24	0.7228	0.6249	0.4097
	Pine	8.11	7.85	9.01	8.84	0.36	0.1424	0.0828	0.6160

^(1)^ Extract concentrations are based on the quantity of Timothy Hay (300 mg) substrate. ^(2)^ SEM, Standard error of the mean. ^(3)^ T, Treatments effect; L, Linear effect; Q, Quadratic effect. ^(4)^ Herb: *Mentha canadensis* plant extract. ^(5)^ Pine: *Pinus densiflora* plant extract. ^a–c^ Means with different superscript letters in the same row indicate significant differences (*p* < 0.05).

**Table 6:** Effect of *Pinus densiflora* and *Mentha canadensis* on abundances of bacteria, archaea and protozoa, in 12 and 24 h of in vitro incubation.

Item	Extract Concentration ^(1)^ (mg/L)				SEM ^(2)^	*p* Value ^(3)^		
	0	30	50	70		T	L	Q
***Pinus densiflora (12 h)***								
*Ruminococcus albus*	1.00	1.15	0.93	1.00	0.07	0.1930	0.5809	0.3892
*Fibrobacter succinogenes*	1.00 ^a^	0.85 ^a^	0.27 ^b^	1.02 ^a^	0.09	0.0010	0.1474	0.0025
*Ruminococcus flavefaciens*	1.00 ^b^	0.86 ^b^	1.46 ^a^	0.93 ^b^	0.07	0.0032	0.2266	0.0945
*Ciliate associated methanogen*	1.00 ^b^	1.42 ^b^	3.06 ^a^	2.03 ^ab^	0.35	0.0165	0.0155	0.1927
***Pinus densiflora (24 h)***								
*Ruminococcus albus*	1.00	1.07	0.88	1.09	0.09	0.3989	0.8606	0.5979
*Fibrobacter succinogenes*	1.00 ^b^	1.31 ^b^	0.45 ^c^	2.18 ^a^	0.17	0.0020	0.0143	0.0085
*Ruminococcus flavefaciens*	1.00	0.93	1.36	1.11	0.11	0.1553	0.1643	0.7306
*Ciliate associated methanogen*	1.00	0.83	1.16	1.22	0.23	0.6440	0.4032	0.5144
***Mentha Canadensis (12 h)***								
*Ruminococcus albus*	1.00	1.17	1.22	1.03	0.13	0.6194	0.7263	0.2397
*Fibrobacter succinogenes*	1.00 ^a^	0.88 ^a^	0.17 ^b^	0.94 ^a^	0.06	0.0001	0.0061	0.0004
*Ruminococcus flavefaciens*	1.00	1.02	1.35	0.95	0.14	0.2357	0.7238	0.2457
*Ciliate associated methanogen*	1.00 ^b^	1.27 ^b^	3.39 ^a^	1.60 ^b^	0.26	0.0008	0.0083	0.0192
***Mentha Canadensis (24 h)***								
*Ruminococcus albus*	1.00	1.06	0.99	1.2	0.0766	0.2605	0.1699	0.3500
*Fibrobacter succinogenes*	1.00 ^a^	0.93 ^a^	0.15 ^c^	0.49 ^b^	0.0691	0.0003	0.0003	0.2000
*Ruminococcus flavefaciens*	1.00	1.16	2.39	1.34	0.202	0.0050	0.0313	0.0500
*Ciliate associated methanogen*	1.00 ^b^	0.82 ^b^	1.12 ^a^	0.92 ^b^	0.2288	0.8152	0.9909	0.9500

^(1)^ Extract concentrations are based on quantity of Timothy Hay (300 mg) substrate. ^(2)^ SEM, Standard error of the mean. ^(3)^ T, Treatments effect; L, Linear effect; Q, Quadratic effect. ^a–c^ Means with different superscript letters in the same row indicate significant differences (*p* < 0.05).
